# Response of Arbuscular Mycorrhizal Fungi to Hydrologic Gradients in the Rhizosphere of *Phragmites australis* (Cav.) Trin ex. Steudel Growing in the Sun Island Wetland

**DOI:** 10.1155/2015/810124

**Published:** 2015-06-03

**Authors:** Li Wang, Jieting Wu, Fang Ma, Jixian Yang, Shiyang Li, Zhe Li, Xue Zhang

**Affiliations:** State Key Lab of Urban Water Resource and Environment, School of Municipal and Environmental Engineering, Harbin Institute of Technology, Harbin 150090, China

## Abstract

Within the rhizosphere, AM fungi are a sensitive variable to changes of botanic and environmental conditions, and they may interact with the biomass of plant and other microbes. During the vegetative period of the *Phragmites australis* growing in the Sun Island Wetland (SIW), the variations of AM fungi colonization were studied. Root samples of three hydrologic gradients generally showed AM fungi colonization, suggesting that AM fungi have the ability for adaptation to flooded habitats. There were direct and indirect hydrological related effects with respect to AM fungi biomass, which interacted simultaneously in the rhizosphere. Though water content in soil and reed growth parameters were both positively associated with AM fungi colonization, only the positive correlations between reed biomass parameters and the colonization could be expected, or both the host plant biomass and the AM fungi could be beneficial. The variations in response of host plant to the edaphic and hydrologic conditions may influence the effectiveness of the plant-mycorrhizal association. This study included a hydrologic component to better assess the role and distribution of AM fungi in wetland ecosystems. And because of that, the range of AM fungi was extended, since they actually showed a notable adaptability to hydrologic gradients.

## 1. Introduction 

Interactions between plants and their rhizosphere microorganisms can significantly affect the corresponding ecosystem function. One key microbial component of the rhizosphere is the arbuscular mycorrhizal fungi (AM fungi), which can form symbiotic relationships with the majority of terrestrial plant roots [1]. These ubiquitous fungi are grouped into the phylum* Glomeromycota*. They can form living root-soil links and a specific zone of soil, which is called mycorrhizosphere [2]. The AM fungi can have an effect on rhizosphere through various mechanisms, such as alterations in soil properties, microbial community, and/or root exudates [3–5]. The symbiosis may help plants to thrive by colonizing a wide soil volume, accelerating photosynthesis, protecting plants against plant pathogens and pests in soil, absorbing resources efficiently, and dissipating of pollutants from the soil [4, 6–8]. They also have the ability for adaptation to different conditions and being synergistic with indigenous soil microorganisms [9]. It has also been proposed that AM fungi can increase the solubility of some immobile nutrients by releasing certain enzymes [10].

AM fungi can form symbiotic relationships with the majority of terrestrial plant biomass. But because the soils of wetlands are often saturated and subsequently lack available oxygen for aerobic soil microorganisms, AM fungi were historically thought to be rare in wetland ecosystem [1, 11]. As a result, although the effects of AM fungi on plant and soil in terrestrial ecosystems are well known [12, 13], these fungi in aquatic and wetland habitats have gotten little attention [14, 15]. Recently, an increasing number of studies have revealed that AM fungi exist in wetland habitats [16, 17]. Stevens et al. [17] found AM fungi colonization in 31 plant species in a bottomland hardwood forest. Besides, several wetland plant species that were thought to be nonmycorrhizal have been found to have high levels of AM fungi colonization [18]. It is now recognized that AM fungi are prevalent in wetlands [14]. It has been also suggested that the success of ecosystem reforestation efforts is likely to depend on the establishment of mycorrhizas, and AM fungi should receive special attention in indigenous plant biomass production and restoration [19]. However, the factors which affect the levels of AM fungi colonization and the relationships between plant biomass, native rhizospheric microorganism communities, and AM fungi in wetland habitats are poorly understood [15].

Because* Phragmites australis* (Cav.) Trin ex. Steudel is a widespread helophyte characteristic of the ecotone between terrestrial and aquatic environments in freshwater to brackish water bodies [20], exhibiting a wide tolerance to the conditions [21], especially to water depth [22], it was chosen as the object plant of this research. AM fungi have been reported on reed [1], and one of the important reasons may be that reed can vent its underground tissues [23]. However, previous researchers have rarely clarified the relationship between AM fungi and reed establishment in wetlands and investigated whether this symbiotic phenomenon depended on the habitat conditions [24]. Though these studies differ greatly with respect to sampling time and venue, it is still difficult to identify which is the primary factor influencing the patterns of AM fungi colonization across different hydrologic gradients, because the fungi in aquatic and wetland habitats have been paid little attention for the reasons mentioned above. Therefore, the specific aims were (1) to assess the variations of hydrologic gradients in the relationships among AM fungi, reed, and rhizospheric microorganisms; (2) to investigate the possible factors that affect AM fungi colonization and determine the primary one among them. The main findings could shed some light on the mechanisms inside AM fungi-reed symbioses and would be referred to optimize the application of phyto-rhizoremediation.

## 2. Materials and Methods

### 2.1. Sample Collection and Analysis

The Sun Island Wetland (SIW) is located at 126°31′–126°36′E, 45°41′–45°47′N on the north shore of Songhua River (Harbin, China). SIW is in the temperate continental monsoon climate zone. During June (summer) and October (autumn) of both 2010 and 2011, the mean daily air temperatures ranged from 16.0 to 26.0°C in June and from 2.6 to 11.2°C in October, while the mean daily relative humidity ranged from 46.7 to 88.7% in June and from 48.2 to 80.7% in October. The Sun Island Wetland is in a triple functional zone overlapped by urbanization areas, development zones, and scenic spots [25].

Three sampling areas were chosen along the hydrologic gradients of SIW (about 50 meters apart). Point SIW1 is flooded with water, the reeds growing in this area are frequently submerged to a depth of up to 15 cm and the soil is always waterlogged. Point SIW2 is located in the river bank, where flooding rarely occurs but where the soil is frequently waterlogged during the wet period. Point SIW3, only water saturated, is located at the outermost bank of the river, where the reeds still have a sufficient water supply from the river during the dry period. During the summer and autumn of 2010-2011, ten samples (including reed plant and rhizospheric soil) were selected from each of the three sampling areas randomly, and each sample was collected within a plot (about 30 × 30 × 30 cm^3^).

Sampling was conducted during 2010 and 2011, the samples were analyzed immediately after they were collected and the backup samples were temporarily stored in a refrigerator to keep them fresh. Each rhizospheric soil sample was divided into 3 parts; one part was stored in 4°C to keep fresh for Biolog, the second part was stored in −20°C for DGGE, and the third part was dried to a constant weight for element analysis. Each plant sample was divided into two parts; one part was stored in refrigerator temporarily to keep fresh and the other part was dried to a constant weight for element analysis. Fine fresh roots were carefully separated from the soil and fixed in ethanol for later studies of AM fungi colonization. The organic matter content of soil was determined by the wet combustion method [26]. The content of organic C was determined by Total Organic Carbon Analyzer (SSM-5000A; Shimadzu Corp., Kyoto, Japan). The content of total N and total S in the soil was determined by a Carbon/Nitrogen/Oxygen/Sulphur Analyzer (Vario EL; Elementar Analysensysteme GmbH, Hanau, Germany). The dried samples were homogenized and subsequently mineralized with HNO_3_ (67%)-HCl (30%)-HF (49%) acids (5 : 2 : 2, V/V/V) in Microwave Digestion System (MARS-5; CEM, Matthews, North Carolina), and then the mineralized samples were analyzed for total P, K, Ca, and Mg, using Inductively Coupled Plasma-Atomic Emission Spectrometry (ICP-AES) (Perkin Elmer Optima 5300DV, Waltham, Massachusetts).

### 2.2. Rhizospheric Microbial Characteristics Analysis

Community level physiological profiles (CLPPs) were assessed by the Biolog EcoPlateTM system (Biolog Inc., CA, USA) as described by Gomez et al. [27]. The color development in each well was recorded as optical density (OD) at 590 nm and 750 nm with a plate reader at regular 24-hour intervals [28]. All work during plate preparation was done under a laminar-flow hood to minimize the risk of contamination.

Microbial activity in each microplate, expressed as average well-color development (AWCD), was determined as follows [27]:

(1) $$\begin{array}{c} AWCD=\frac{\sum {OD}_{i}}{31}, \end{array}$$

where OD_*i*_ is the optical density value from each well [28].

The Shannon-Weaver diversity index (*H*) and richness index (*S*) were calculated using an OD of 0.25 as threshold for positive response, which was described by Garland and Derry et al. [29, 30]:

(2) $$\begin{array}{c} H=-\sum p_{i}\left(\ln p_{i}\right), \end{array}$$

where *p*_*i*_ is the ratio of the activity on each substrate to the sum of activities on all substrates.

The DGGE profiles were assessed by the DCode system (BioRad Co., Ltd., USA). The DNA of rhizospheric microbes was extracted with a FastDNA Spin Kit for Soil (Q-Biogene, Vista, CA, USA). The extracted DNA was used as a template for PCR. The primers of bacteria, actinomycetes, and fungi for the PCR amplification were designed, respectively, and the corresponding thermocycling conditions were set. Genes of bacteria were amplified with primers GC-341F (5′-CCTACGGGAGGCAGCAG-3′) and 534R (5′-ATTACCGCGGCTGCTGG-3′). The thermocycling conditions were (touchdown PCR) 3 min at 95°C, followed by 20 cycles of 30 s at 95°C (annealing for 30 s with a 0.5°C/cycle decrement until the 56°C is reached), 1 min at 72°C, 35 cycles of 30 s at 95°C and 30 s at 56°C and 1 min at 72°C, and a final extension for 5 min at 72°C [31, 32]. Genes of fungi were amplified with primers GC-FR1 (5′-AICCATTCAATCGGTAIT-3′) and FF390 (5′-CGATAACGAACGAGACCT-3′). The thermocycling conditions were 8 min at 95°C, followed by 30 cycles of 30 s at 95°C and 45 s at 50°C, 2 min at 72°C, and a final extension for 10 min at 72°C [33]. Electrophoresis was performed in a DCode system (Bio-Rad Co., Ltd., Hercules, California). The DGGE profiles were analyzed by software “Quantity One version 4.6.2” (BIO-RAD Laboratories, Inc., USA).

The Shannon-Weaver diversity index (*H*) and richness index (*S*) were determined according to the following equation as described by Yang et al. [34]:

(3) $$\begin{array}{c} H=-\sum p_{i}\left(lnp_{i}\right)=-\sum \left(\frac{N_{i}}{N}\right)ln\left(\frac{N_{i}}{N}\right), \end{array}$$

where *p*_*i*_ is the percentage of the DGGE band gray degree to each DNA sample, *N*_*i*_ is the net gray degree quantity (subtracted by the background gray degree quantity of a gel) of the DGGE band to each DNA sample, *N* is the total net gray degree quantity, and *S* is the number of DGGE bands to each DNA sample (richness index).

### 2.3. Assessment of AM Fungi Colonization

Samples of fine roots were cleared in 10% w/v KOH and stained with 0.5% acid fuchsin as described by Li et al. [35]. Root colonization described as the percentage of root length with hyphae or vesicles was estimated using a line intercept approach and determined using procedures described by McGonigle et al. [36]. Root segments were examined under a microscope (Olympus CX31, Olympus). For assessment of AM colonization levels, the variables considered to characterize AM colonization were the percentages of arbuscules (*A*%), hyphae (*H*%), vesicles (*V*%), mycorrhizal frequency (*F*%), and mycorrhizal intensity (*M*%) [37]. Hyphae were only scored if attached to other AM fungi structures [38]. Thirty root fragments of each plant individuals were used to estimate AM fungi colonization parameters of reed.

### 2.4. Statistics and Data Analyses

Standard error (SD) was used as a measure of variance. One-way ANOVA (Duncan test) was performed to ascertain whether parameters were significantly different among treatments (*α* = 0.05). The bivariate correlations (Pearson correlation coefficient) were performed, via using SPSS Statistical Software Package (version 17.0) (SPSS Inc., Chicago, Illinois) for Windows.

## 3. Results

**Figure 1:**
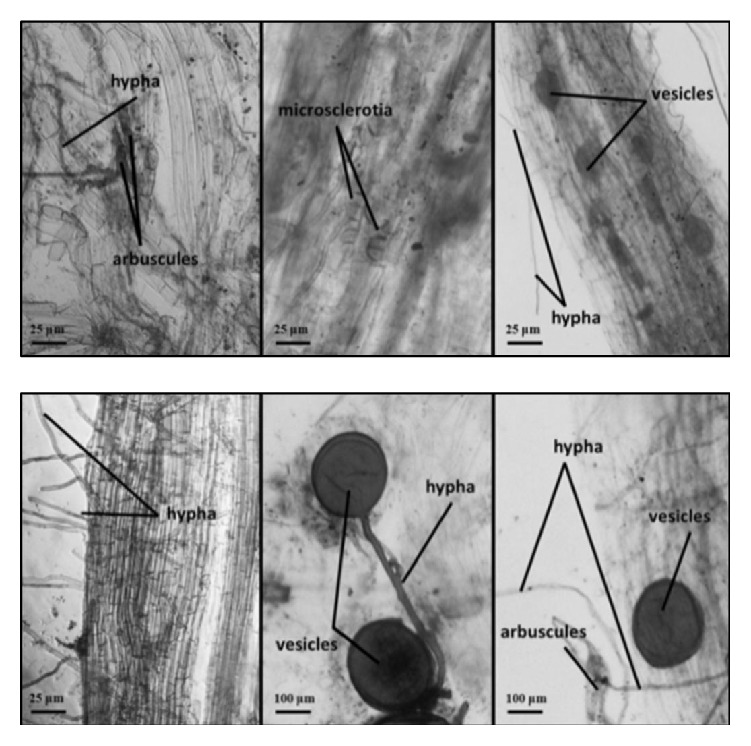
Arbuscular mycorrhizal structures in the roots of* Phragmites australis*.

AM fungi colonization was observed through a microscope (Figure 1). Regardless of the hydrologic gradient, mean values for the proportion of root segment colonized by hypha ranged from 13% to 20%, which were less variable than the vesicle colonization (ranged from 2% to 6%) and the arbuscules colonization (ranged from 2% to 8%) (Table 1). Despite the AM fungi structures (hypha, vesicle, and arbuscules) intensity in roots of* Ph. australis* was not high, it showed a tendency to change along with the hydrologic gradient, with the general order: SIW1 > SIW2 > SIW3. The AM fungi hypha colonization varied significantly between SIW1 and SIW3 (autumn), but no significant difference was found between SIW1 and SIW2 (autumn). In contrast, the AM fungi arbuscules colonization varied significantly between SIW1 and SIW3 (summer), but no significant difference was found between SIW1 and SIW2 (summer). In addition, the frequency and intensity of AM fungi colonization also varied significantly between SIW1 and SIW3 both in summer and autumn, but there were no significant differences between SIW1 and SIW2 (summer).

Correlation analyses were performed between the AM fungi colonization and the rhizosphere soil physicochemical properties of* Ph. australis* (Table 2). Note that the moisture content had significant positive relationships with arbuscular mycorrhizal fungi colonization (hyphae, vesicles and the mycorrhizal frequency). In contrast, pH, organic carbon, total N, total P, total K, and Ca had significant negative relationships with arbuscular mycorrhizal fungi colonization. In addition, compared to the arbuscular mycorrhizal structures (hyphae, vesicles, and arbuscules), the frequency and intensity have relationships with more physicochemical properties.

Correlation analyses were also performed between the AM fungi colonization and the characteristics of the rhizospheric microbial community of* Ph. australis* (Table 3). Note that the rhizospheric microbial biomass had significant negative relationships with the frequency and intensity of arbuscular mycorrhizal fungi colonization, especially the arbuscules. In contrast, hyphae and vesicles had no significant negative relationships with arbuscular mycorrhizal fungi colonization. Besides, compared to the frequency and intensity, the arbuscules have relationships with more physicochemical properties.

In addition, correlation analyses were performed between the AM fungi colonization and the growth parameters of* Ph. australis* (Table 4). The results indicated that the biomass of* Ph. australis* had significant positive relationships with the arbuscules, the arbuscular mycorrhizal frequency and intensity. In contrast, hyphae and vesicles had no significant relationships with arbuscular mycorrhizal fungi colonization. Besides, the frequency and intensity have relationships with all the related physicochemical properties.

## 4. Discussion

AM fungi have the ability for adaptation to different conditions [9]. The variations of hydrologic gradients in the relationships among the components of reed rhizosphere were studied, which showed that AM fungi were present along hydrologic gradients in the wetland of Northeast China. The maximum colonization occurred at the sampling point with the highest moisture content (SW1) and was minimal in the rhizosphere with the lowest moisture content (SWA3), which suggested that AM fungi not only have the ability for adaptation to aquatic and wetland habitats, but also have preferential colonization with respect to hydrologic gradient. Observed variations in the dynamics of different AM fungi structures were significant in this research. Previous studies proved that AM fungi dynamics are strongly affected by their capacity to colonize in roots and scavenge carbohydrates and minerals, respectively. These direct and indirect effects with respect to AM fungi interact simultaneously in the rhizosphere [39]. Consequently, we hypothesize that both habitat condition (living matter and nonliving matter) and host plant growth can affect the dynamics of AM fungi. However, we still need to know whose effect is greater.

One of the possible factors affecting AM fungi colonization was water level changing. However, there is no consensus about the effect of moisture content on AM fungi growth. The effect of increase in moisture content in soil on AM fungi colonization has been generally documented: when plants become submerged, a decrease in AM fungi colonization is usually found [1], because the saturated soil subsequently lacks available oxygen for aerobic soil microorganisms, such as AM fungi [11]. But some studies proved that there are no relationships between AM fungi colonization and moisture content [40]. As reported by some researchers, once AM fungi symbiosis was established, subsequent increase in water level or even permanent flooding did not affect their colonization in the roots [1, 41]. In addition, although the growth of external mycelium of AM fungi is thought to be improved by organic matter content [42], the carbon incorporated into AM fungi biomass actually originates from plant photosynthates rather than the surrounding organic matter [43]. But the organic matter could still act as an important source of other nutrients, such as P, for AM fungi [44]. The results suggested that soil organic matter content maybe not the principal promoter of AM fungi colonization. In addition, the trend that moisture content in soil was positively associated with AM fungi root colonization probably could explain the fact that AM fungi growth was suppressed by certain environmental factors which were decreasing with moisture content, such as P. P was proved to have negative effects on AM fungi growth [45, 46]. In such an intermittent ecosystem, the amount of plant available nutrients depends on soil moisture and oxygen availability, which may disguise the relationships between soil properties and AM fungi colonization [1]. Moreover, the AM fungi biomass could also affect the properties of rhizosphere through indirect mechanisms including altering soil acidity [3] and producing more root exudates [47]. Thus, we still requires more details to determine how the variations of soil parameters and water level combinedly affect AM fungi colonization on the Sun Island Wetland.

Moisture content in soil was associated with AM fungi colonization, which also suggested that moisture content possibly affected AM fungi through changing their symbiotic partners: the indigenous rhizospheric microorganisms and host plant. Therefore, another possible factors affecting AM fungi colonization could be rhizospheric microorganisms. The plant rhizosphere is a dynamic complex system in which many parameters may influence the population structure, diversity, and activity of the microbial community [48]. Therefore, the interactions between AM fungi and other indigenous soil organisms are complex. The reciprocal effect of AM fungi and rhizospheric microorganisms has also been widely examined, but principally using in vitro systems [49, 50]. For instance, Filion et al. [51] showed that exudates from the hyphae of* Glomus intraradices* could stimulate the growth of certain microorganisms on agar. In this study, DGGE and Biolog were used to assess the microbial community characteristics in the rhizosphere of reed in different ways. It is well known that AM fungi are synergistic with mycorrhizal helper bacteria and plant growth-promoting rhizobacteria [52, 53]. Though beneficial interactions have been frequently mentioned [54], other evidence has also suggested antagonistic interactions with bacteria, fungi, and microarthropods that may affect the functioning of the AM fungi symbioses [55–57]. We expected to find that the AM fungi influenced the indigenous microbial community and benefited from it. However, the results did not support this synergistic relationship. What we found was that AM fungi growth was seemly reduced by certain indigenous soil microorganisms and the presence of AM fungi did not increase the diversity and richness of the microbial community. Several authors also reported that AM fungi may play a role in controlling soil microbial communities [58]. Either the competition for resources between AM fungi and other microorganisms or the suppressive effects of certain types of microorganisms on AM fungi growth may lead to reduction in the growth of AM fungi. Competition between AM fungi and soil microorganisms may attribute to the so-called Gadgil effect [59]. Leigh et al. [60] found that the absence of live bacterial inoculums could increase the P concentration in AM fungi hyphae colonized root, because the competition between AM fungi and bacteria inhibited the ability of AM fungi to acquire resources directly from organic matter. Other researchers also suggested that the effects of microbial community on AM fungi growth and function were greater than the reciprocal impact [60]. de Jaeger et al. [61] demonstrated that* Trichoderma harzianum* was able to impact the viability of AM fungi by feeding on its intra- and extraradical mycelium under in vitro controlled conditions. In natural ecosystems, the rhizosphere effects may be the dominant influence [62, 63]. However, there is no consensus about the effect of microbial communities on AM fungi growth [64].

The lack of positive correlation between AM fungi colonization and the habitat (living and nonliving matter) of* Ph. australis* rhizosphere was possibly due to the fact that plant growth exerted relatively dominant effect on mycorrhizal colonization. The results suggest that more vigorous plants could maintain higher AM fungi colonization rate. Since* Ph. australis* is a kind of well-adapted helophyte to water level fluctuation, the phenomenon that AM fungi colonized reed under flooded conditions is not illogical. Their ability to survive in such conditions is mainly due to the aerenchyma in the stems and roots, through which the host plants can ventilate their own underground tissues by pressurized gas flow [23]. From this perspective, the more vigorously host plants grow, the better their stems and roots develop. As a result, the more oxygen can be delivered to promote the AM fungi dynamics in the root system. Vice versa, the results also suggest the converse that AM fungi colonization could promote plant growth. A positive in situ correlation between reed biomass and AM fungi can be expected in this research, but whether the enhanced plant sturdiness is the consequence or the promoter of AM fungi colonization is difficult to establish. AM fungi can form symbiotic associations with the roots of most terrestrial plants and provide many benefits including improved nutrient uptake, flood and drought resistance, and herbivore resistance [13, 65]. It is well known that arbuscules are the major site for the transfer of minerals and carbohydrates between both partners of the symbiosis [13]. Because AM fungi can promote decomposition of organic material [66] and acquire substantial amounts of N that can be transferred to plant partners [67], AM fungi are antagonistic to pathogenic microorganisms and synergistic with plant growth-promoting rhizobacteria [53, 68]. The effects of AM fungi on biomass of various plant species have been reported mainly in studies under experimental conditions with controlled environmental and soil parameters [41, 69, 70]. Field studies that have focused on the benefits of AM fungi for plant biomass are rarely documented [71]. Therefore, further studies should be conducted to ascertain these results under controlled conditions.

In conclusion, this study included hydrologic components to better assess the dynamics, distribution, and role of AM fungi in wetland ecosystems. Although moisture content in soil and reed growth parameters were both positively associated with AM fungi colonization, only the correlation between reed biomass and AM fungi can be expected. Since AM fungi showed a response to the conditions of their host plant and performed as a tie of the tripartite correspondence between the symbiotic partners of plant biomass and rhizospheric microbial biomass, its application as a biomonitor should be considered in further research.

## Figures and Tables

**Table 1:** Characteristics of arbuscular mycorrhizal fungi colonization of *Phragmites australis.* Area SW1 is flooded with water sometimes; the reeds growing in this area are frequently submerged to a depth of up to 15 cm and the soil is always waterlogged. Area SW2 is located in the river bank, where flooding rarely occurs but the soil is always saturated during the wet period. Area SW3, only humid, is located at the outermost bank of the river, where the reeds still have a sufficient water supply from the river during the dry period. S: summer; A: autumn.

	S			A		
	SIW1	SIW2	SIW3	SIW1	SIW2	SIW3
Hyphae	19.2 ± 3.56^A^	17.7 ± 2.18^A^	13.2 ± 2.19^A^	21.6 ± 3.51^b^	16.6 ± 2.34^b^	9.3 ± 2.2^a^
Vesicles	5.6 ± 0.65^B^	3.7 ± 0.45^A^	2.9 ± 0.29^A^	4.7 ± 0.59^c^	3.6 ± 0.53^b^	2.3 ± 0.38^a^
Arbuscules	3.5 ± 0.35^B^	2.9 ± 0.3^AB^	2.7 ± 0.35^A^	7.9 ± 1.1^a^	7.2 ± 1.33^a^	6.3 ± 0.9^a^
Frequency	26.3 ± 2.38^B^	23.2 ± 3.64^B^	16.1 ± 2.7^A^	33.2 ± 4.92^b^	27.2 ± 4.29^a^	16.9 ± 1.99^a^
Intensity	7.4 ± 0.62^B^	6.3 ± 0.95^AB^	5.4 ± 0.78^A^	11.7 ± 2.5^b^	9.2 ± 1.76^a^	5.5 ± 0.91^a^

Different letters in uppercase indicate significant difference between three hydrologic gradients in summer (S) (*α* = 0.05) after one-way ANOVA (Duncan test). Different letters in lowercase indicate significant difference between three hydrologic gradients in autumn (A) (*α* = 0.05) after one-way ANOVA (Duncan test). Data are mean ± SD (*n* = 30).

**Table 2:** Coefficients of Pearson's correlations between the characteristics of arbuscular mycorrhizal fungi colonization and the rhizosphere soil physicochemical properties of *Phragmites australis* (MC: moisture content; OM: organic matter; OC: organic carbon).

	pH	MC (%)	OM (%)	OC (%)	N (mg kg^−1^)	P (mg kg^−1^)	S (mg kg^−1^)	K (mg kg^−1^)	Ca (mg kg^−1^)	Mg (mg kg^−1^)
SIW1	8.23	28.13	15.73	13551	957	413.3	396.7	20523	12145	992
SIW2	8.26	20.87	16.69	15327	1065	432.1	287.9	22367	14003	1132
SIW3	8.28	12.52	12.24	18256	1193	481.2	271.1	23579	13795	1171
AIW1	7.69	27.51	13.52	11095	836	262.9	357.3	19333	8753	1272
AIW2	7.71	23.01	13.75	12337	893	331.9	230.1	20471	8886	1333
AIW3	7.76	11.05	11.86	14511	997	396.7	219.9	23451	11795	1354

Hyphae	ns	∗∗	ns	ns	ns	ns	ns	ns	ns	ns
Vesicles	ns	∗∗	ns	ns	ns	ns	ns	ns	ns	ns
Arbuscules	−^**^	ns	ns	ns	ns	ns	ns	ns	−^*^	ns
Frequency	ns	∗∗	ns	−^*^	−^*^	−^*^	ns	−^*^	ns	ns
Intensity	ns	ns	ns	−^*^	−^*^	−^*^	ns	−^*^	−^*^	ns

ns: no significant correlation at the 0.05 level (2-tailed).

^*^Correlation is significant at the 0.05 level (2-tailed).

^**^Correlation is significant at the 0.01 level (2-tailed).

**Table 3:** Coefficients of Pearson's correlations between the characteristics of arbuscular mycorrhizal fungi colonization and the characteristics of the rhizospheric microbial community of *Phragmites australis*. ((*H*): Shannon-Weaver diversity index; (*S*): Shannon-Weaver richness index; B: bacteria; A: actinomycetes; F: fungi).

	Genetic characteristics				Metabolic characteristics		
	(*H*)-B	(*H*)-F	(*S*)-B	(*S*)-F	AWCD	(*H*)	(*S*)
SIW1	3.2	2.91	25	19	0.64	2.62	3.2
SIW2	3.45	3.06	32	22	0.67	2.89	3.45
SIW3	3.46	3.1	32	23	0.65	2.73	3.46
AIW1	2.98	2.18	20	9	0.57	2.29	2.98
AIW2	3.08	2.47	22	12	0.63	2.55	3.08

Hyphae	ns	ns	ns	ns	ns	ns	ns
Vesicles	ns	ns	ns	ns	ns	ns	ns
Arbuscules	−^**^	−^*^	−^**^	−^*^	−^*^	−^*^	−^*^
Frequency	ns	−^*^	ns	−^*^	ns	ns	ns
Intensity	ns	−^**^	ns	−^**^	ns	ns	ns

ns: no significant correlation at the 0.05 level (2-tailed).

^*^Correlation is significant at the 0.05 level (2-tailed).

^**^Correlation is significant at the 0.01 level (2-tailed).

**Table 4:** Coefficients of Pearson's correlations between the characteristics of arbuscular mycorrhizal fungi colonization and the growth parameters of *Phragmites australis. *(ShL: shoot length; BD: basal diameter; LA: leaf area; RaL: rachis length; ShW: shoot dry weight).

	ShL (cm)	BD (mm)	LA (cm^2^)	RaL (cm)	ShW (g)
SIW1	174.37	8.73	325.62	21.3	8.49
SIW2	128.36	8.43	301.35	18.2	7.09
SIW3	116.35	7.73	171.03	15.6	5.69
AIW1	265.49	9.47	399.24	27.2	19.06
AIW2	225.46	8.93	382.32	25.2	15.55
SIW1	207.51	8.53	260.22	22.9	11.38

Hyphae	ns	ns	ns	ns	ns
Vesicles	ns	ns	ns	ns	ns
Arbuscules	∗∗	ns	ns	ns	∗∗
Frequency	∗	∗	∗∗	∗∗	∗
Intensity	∗	∗	∗	∗∗	∗

ns: no significant correlation at the 0.05 level (2-tailed).

^*^Correlation is significant at the 0.05 level (2-tailed).

^**^Correlation is significant at the 0.01 level (2-tailed).
